# The relationship between the phosphate and structural carbonate fractionation of fallow deer bioapatite in tooth enamel

**DOI:** 10.1002/rcm.8324

**Published:** 2018-12-19

**Authors:** Holly Miller, Carolyn Chenery, Angela L. Lamb, Hilary Sloane, Ruth F. Carden, Levent Atici, Naomi Sykes

**Affiliations:** ^1^ Department of Archaeology University of Nottingham Nottingham NG7 2RD UK; ^2^ NERC Isotope Geosciences Facility, Kingsley Dunham Centre, British Geological Survey Keyworth, Nottingham NG12 5GG UK; ^3^ Adjunct Research Fellow, School of Archaeology University College Dublin Belfield Dublin 4 Ireland; ^4^ Department of Anthropology University of Nevada Las Vegas, Box 455003 4505 S. Maryland Parkway Las Vegas NV 89154‐5003 USA; ^5^ Department of Archaeology University of Exeter Laver Building Streatham Campus Exeter EX4 4Q UK

## Abstract

**Rationale:**

The species‐specific relationship between phosphate (δ^18^O_P_ values) and structural carbonate (δ^18^O_C_ values) oxygen isotope ratios has been established for several modern and fossil animal species but until now it has not been investigated in European fallow deer (*Dama dama dama*). This study describes the relationship between phosphate and structural carbonate bioapatite in tooth enamel of extant fallow deer, which will help us further understand the species' unique environmental and cultural history.

**Methods:**

The oxygen isotope composition of phosphate (δ^18^O_P_ value) and structural carbonate (δ^18^O_C_ value) of hydroxylapatite was determined in 51 modern fallow deer tooth enamel samples from across Europe and West Asia. The δ^18^O_C_ values were measured on a GV IsoPrime dual‐inlet mass spectrometer and the δ^18^O_P_ values on a temperature‐controlled elemental analyser (TC/EA) coupled to a DeltaPlus XL isotope ratio mass spectrometer via a ConFlo III interface.

**Results:**

This study establishes a direct and linear relationship between the δ^18^O_C_ and δ^18^O_P_ values from fallow deer tooth enamel (δ^18^O_C_ = +9.244(±0.216) + 0.958 * δ^18^O_P_ (±0.013)). Despite the successful regression, the variation in δ^18^O values from samples collected in the same geographical area is greater than expected, although the results cluster in broad climatic groupings when Koppen‐Geiger classifications are taken into account for the individuals' locations.

**Conclusions:**

This is the first comprehensive study of the relationship between ionic forms of oxygen (phosphate oxygen and structural carbonate) in fallow deer dental enamel. The new equation will allow direct comparison with other herbivore data. Variable δ^18^O values within populations of fallow deer broadly reflect the ecological zones they are found in which may explain this pattern of results in other euryphagic species.

## 1 INTRODUCTION

A recent programme of inter‐disciplinary research has shown that the distribution of fallow deer (*Dama dama dama*) is a direct record of human migration, trade, behaviour and worldview.^1^, ^2^, ^3^, ^4^ Prior to this understanding of the cultural significance of fallow deer, the species has been under‐investigated in favour of the red (*Cervus elaphus*) and roe (*Capreolus capreolus*) deer, i.e. those that are native to western Europe. Yet the very presence of fallow deer in areas beyond their native eastern Mediterranean habitat, transported and maintained by human groups, demonstrates that investigations of their habits, habitats and provenance are important to palaeoenvironmental, human‐animal and archaeological studies.

Strontium isotope analysis has been used to great effect to infer ancient translocations and source populations of fallow deer;^2^, ^3^ however, these issues could be addressed with greater resolution if they were complemented by oxygen isotope analysis. As first proposed by Longinelli,^5^ an important application of oxygen isotope biochemistry is paleoclimate reconstruction from fossil bone and tooth enamel. This technique has been used to examine place of origin for humans and animals in the archaeological record, as the isotope signature of local water sources is preserved at the point of mineralisation in dental enamel.^6^, ^7^, ^8^, ^9^

Within mammalian tissues including teeth, antler, ivory, and bone, bioapatite [generalised as Ca_10_(PO_4_,CO_3_)_6_(OH,CO_3_)_2_] contains two ionic forms of oxygen suitable for isotope analysis: structural carbonate (CO_3_^2−^) and the more abundant phosphate (PO_4_^3−^) (hereafter referred to, respectively, as O_C_ and O_P_).^10^, ^11^, ^12^, ^13^, ^14^ Most published studies of bioapatite oxygen measure the δ^18^O_C_ value as it is quicker, easier and cheaper to measure than the δ^18^O_P_ value,^6^, ^15^ and so the relationship between the δ^18^O_P_ and δ^18^O_C_ values has been established for a range of modern and fossil animal species.^15^, ^16^, ^17^, ^18^, ^19^, ^20^, ^21^, ^22^ These data suggest that the relationship between δ^18^O_P_ and δ^18^O_C_ values (slope, intercept and Δ_C‐P_) is species specific,^21^ where Δ_C‐P_ is the difference between the δ^18^O_C_ and δ^18^O_P_ values. Until now this relationship has not been investigated in the widely distributed and culturally important fallow deer.

The isotope ratios of oxygen atoms in O_C_ and O_P_ are cogenetic, as they are formed simultaneously in isotopic equilibrium with body water oxygen. This is directly related to the composition of ingested water, often meteoric water, at a constant body temperature,^5^, ^18^, ^23^, ^24^, ^25^, ^26^ which is sensitive to latitude, altitude and climate.^27^ Thus, if the δ^18^O values of tissues are measured, the results can help to assess an animal's origin and movement.^20^, ^28^, ^29^ The relationship between the δ^18^O_P_ value and the δ^18^O value of drinking water (hereafter the δ^18^O_DW_ value) is also known to be species‐specific and therefore it can also be part of provenance studies.^10^, ^17^, ^18^, ^20^, ^30^ While this is well established in humans^5^, ^23^, ^31^, ^32^ and a range of animals including several deer species (examples in^10^, ^33^), it is currently unknown for fallow deer.

A sample of eight fallow deer from a single area of Italy was included in a larger study of red deer δ^18^O_P_ and δ^18^O_DW_ values by D'Angela and Longinelli,^34^ who concluded that the two species were likely to behave in a similar way. Fallow deer, however, are non‐obligate drinkers, which means that their oxygen isotope ratios will not necessarily reflect local waters. This investigation seeks to clarify whether the relationships between cogenetic δ^18^O_C_ and δ^18^O_P_ values,^15^ and that between δ^18^O_p_ and δ^18^O_DW_ values, can be determined in non‐obligate drinking fallow deer, and therefore to assess the utility of using δ^18^O values of fallow deer in archaeological provenance studies. A further aim of the study is to investigate other factors that may affect the δ^18^O_C_–δ^18^O_P_ relationship in non‐drinking species.

## 2 EXPERIMENTAL

### 2.1 Materials

**Figure 1:**
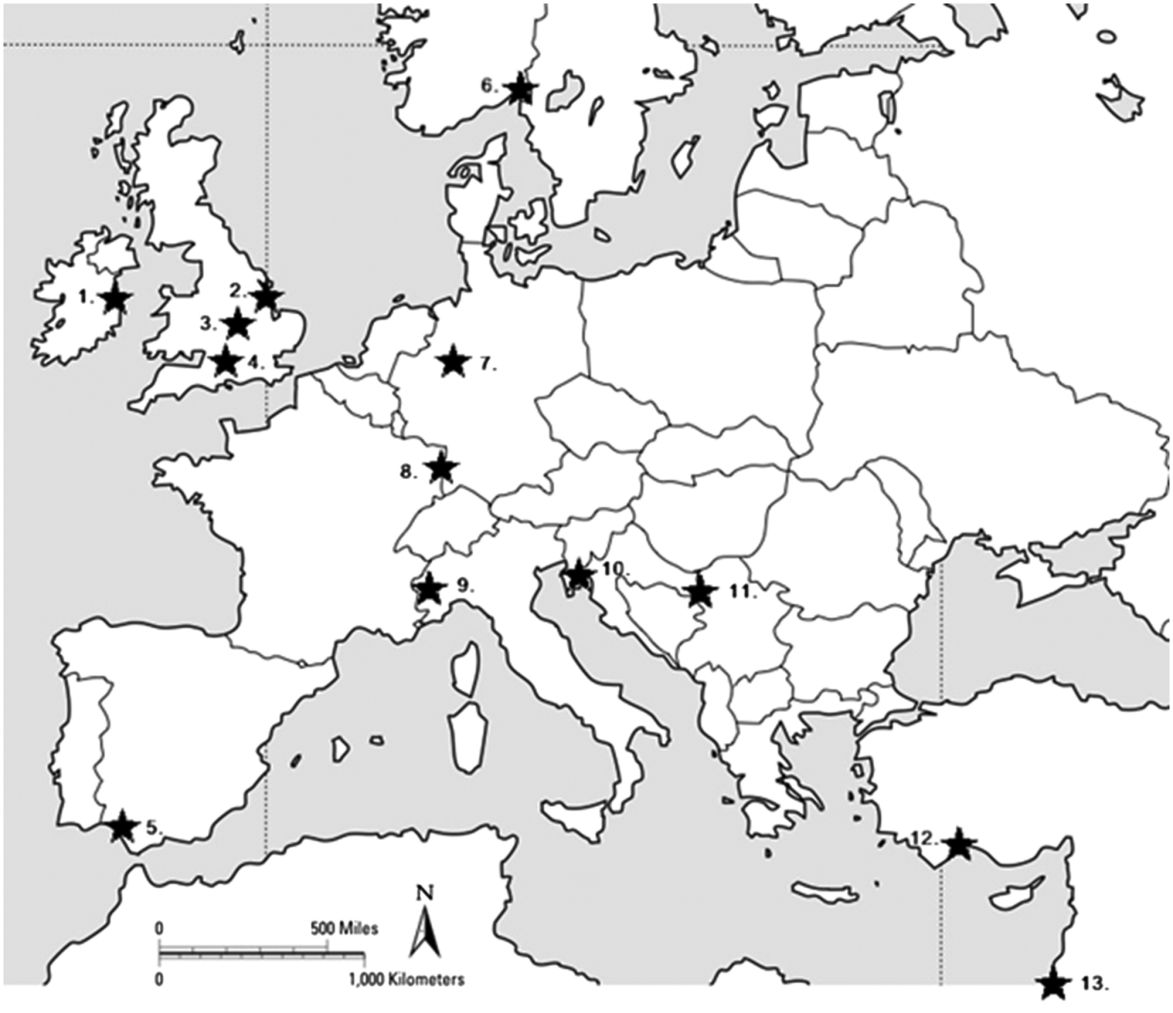
Map showing sample sites for fallow deer populations included in the study

δ^18^O_C_, δ^18^O_P_ and δ^13^C analyses were undertaken on 51 modern fallow deer tooth enamel samples at the NERC Isotope Geosciences Facility (Keyworth, UK). Samples of teeth from different individuals from known fallow deer populations were supplied by volunteers who had access to extant herds across Europe (*n* = 13 locations, see Table 1). As a result, our samples broadly represent the geographic, climate and topographic range of human‐introduced fallow deer distribution across Europe and West Asia.^35^ The distribution of fallow deer has been significantly influenced by humans to the extent that its distribution now spans six continents.^1^, ^2^, ^3^, ^4^, ^35^ In general, when human‐mitigated circumstances allow, they live in a variety of deciduous and mixed woodlands, but rarely thrive at heights of more than 1000 meters above sea level. Fallow deer spend much of their time grazing in nearby fields, keeping close to wooded areas for cover and shelter but also for browse.^35^ As the samples were drawn from herds managed in different ways this study reflects the range of complex and varied relationships that fallow deer have with people in their culturally defined environments. Table 1 and Figure 1 summarise information on the location and circumstances of each of the deer herds.

**Table 1:** Description of sample sites for fallow deer populations included in the study with a description of the local circumstances in which the herds are managed

Map	Location (ref)	Local circumstances of fallow deer herds
1.	Phoenix Park (PP), Ireland	7 km^2^ of urban park environment with flat meadow land
2.	Scrivelsby Park (LN), northern England	Grounds of Scrivelsby Court country estate with *ca*.1 km^2^ of enclosed deer park meadow.
3.	Wytham Woods (WW), central England	4.5 km^2^ of managed woodland and grassland; some areas are fenced but it is mostly open to deer.
4.	Andover (AD), southern England	5.5 km^2^ of open woodland and grassland bisected by the A303 road.
5.	Doñana (SP), Spain	National park of marshland and sand dunes covering 543 km^2^, of which 135 km^2^ is a nature reserve.
6.	Moss (MP), Norway	Southern Jeloy island, Moss. A 19 km^2^ protected landscape of farmland, meadow and mixed forest close to the coast and urban centre (Moss). Deer are free roaming, but excluded from some areas.
7.	Sennelager (GM), Germany	116 km^2^ area of woodland and grassland on a military training ground. The fallow deer are free roaming and known to enter urban areas.
8.	Gerstheim (FR), France	The wild fallow deer were introduced to this area between 1854 and 1858 and roam an area of *ca* 50 km within 650 km^2^ of hunting reserve.
9.	Piedmont (IT), Italy	Free roaming deer hunted in the Grondona area, 26 km^2^ of forest with pasture and urban areas.
10.	Morović (MV), Serbia	25 km^2^ of managed hunting reserve in Srem, Northern Serbia. The area is mainly oak forest and meadows and with rural villages.
11.	Kotredež (SV), Slovenia	An area of central Slovenia. Fallow deer are kept in a 1.96 km^2^ game enclosure of wood and parkland.
12.	Termessos National Park (TK), Turkey	The Düzlerçamı Wildlife Development Area of the Termessos National Park comprises 29,000 km^2^ of forest and open agricultural land of which 4.3 km^2^ is fenced for a fallow deer breeding station.
13.	Haifa (HA), Israel	Zoo population of *Dama dama mesopotamia* kept in a small enclosure in an urban educational zoo setting. Teeth were collected over 2 decades.

### 2.2 Intra‐individual variation

The teeth used in the study were permanent dentition from adult animals. Wherever possible 3^rd^ mandibular molars were collected; however, for three locations (France, Haifa and Turkey) only 1^st^ mandibular incisors were available (Table 2). Fallow deer 1^st^ permanent incisors erupt at 7–8 months and the 3^rd^ molar at 15–26 months.^37^, ^38^ We are aware that this may cause discrepancies in the data presented in this study as inter‐tooth variation has previously been noted in mammalian populations. This is likely to be due to the periods of formation occurring in different seasons and therefore under different climatic circumstances.^39^, ^40^ Further to this, weaning signatures may be preserved in different teeth whereby suckling maternal milk elevates body water δ^18^O values,^41^, ^42^ although Veitschegger and Sánchez‐Villagra^43^ suggest that the permanent dentition used in this study mineralise after the weaning period. To investigate how significant these effects may have been to our study, we have included 1^st^ incisor and 3^rd^ molar pairs from each of four individuals from Phoenix Park to look at the differences between the results from these teeth (Table 2).

**Table 2:** Results for all 51 fallow deer in the study (55 samples in total, including 4 individuals sampled in 3^rd^ molar (M3) and 1^st^ incisor (I)). Column 1 – samples with * indicate those with Δ^18^O‰ <7.3‰. For column 4, Köppen‐Geiger climate classifications,^36^ see Table 3

Sample No	Tooth	**Location (**Figure 1 **ref)**	Environmental/Köppen‐Geiger climate classifications^36^	δ^13^C vPDB	1σ	δ^18^O_C_	1σ	n	δ^18^O_P_	1σ	n	Δ^18^O (δ^18^O_C_ ‐ δ^18^O_P_)	δ^18^O_DW_ (combined deer equation)
ad 1	M3	Andover, UK (4)	Cfb	−15.1	0.12	+27.8	0.11	1	+19.7	0.22	3	8.1	−6.0
ad 2				−14.8	0.12	+25.8	0.11	1	+17.3	0.17	2	8.5	−8.8
ad 3				−16.2	0.12	+26.1	0.11	1	+17.7	0.13	3	8.4	−8.4
FR01	I	Gerstheim, France (8)	Cfb	−17.1	0.12	+20.5	0.11	1	+11.9	0.20	2	8.6	−15.2
FR02 *				−14.5	0.12	+21.7	0.11	1	+15.4	0.11	2	6.3	−11.1
FR03				−18.0	0.12	+20.2	0.11	1	+10.9	0.15	2	9.4	−16.4
GM01	M3	Sennelager, Germany (7)	Cfb	−15.3	0.11	+21.0	0.18	2	+12.6	0.22	2	8.4	−14.4
GM02 *				−16.8	0.12	+20.5	0.11	1	+13.8	0.19	2	6.7	−12.9
GM03				−15.3	0.12	+23.6	0.11	1	+15.5	0.12	4	8.1	−10.9
HA 643 *	I	Haifa, Israel (13)	Csa	−12.7	0.07	+28.6	0.10	2	+21.7	0.03	3	6.9	−3.7
HA 644				−13.7	0.12	+27.8	0.11	1	+18.5	0.13	4	9.3	−7.4
HA 645 *				−14.8	0.12	+27.1	0.11	1	+20.8	0.07	2	6.4	−4.8
IT01	M3	Piedmont, Italy (9)	Cfa	−14.3	0.09	+26.4	0.11	3	+17.8	0.13	3	8.5	−8.2
IT02				−12.5	0.07	+26.6	0.11	4	+18.6	0.27	2	7.9	−7.2
IT03				−13.9	0.12	+28.4	0.06	2	+19.6	0.11	2	8.8	−6.1
LN 01	M3	Scrivelsby Park, UK (2)	Cfb	−16.5	0.12	+26.6	0.11	1	+17.8	0.11	2	8.8	−8.2
LN 02				−17.8	0.07	+23.6	0.15	2	+14.6	0.16	3	9.0	−11.9
LN 03				−17.2	0.12	+25.1	0.11	1	+16.2	0.18	3	8.9	−10.1
MP1 *	M3	Moss Park, Norway (6)	Dfb	−15.7	0.12	+20.9	0.11	1	+14.0	0.00	2	6.9	−12.7
MP2				−15.7	0.04	+21.8	0.18	2	+14.1	0.20	5	7.7	−12.6
MP 3A				−14.4	0.12	+22.3	0.11	1	+13.6	0.23	2	8.6	−13.1
MV 002	M3	Morović, Serbia (10)	Cfb	−12.7	0.22	+26.8	0.14	3	+18.9	0.20	4	7.9	−7.0
MV 003				−14.4	0.09	+27.4	0.14	3	+19.1	0.15	2	8.2	−6.7
MV 004 *				−10.4	0.04	+24.2	0.00	2	+18.3	0.03	2	5.9	−7.6
SP 01	M3	Doñana, Spain (5)	BSk ‐ Csa	−16.6	0.33	+25.7	0.12	2	+17.3	0.14	2	8.5	−8.9
SP 02				−16.4	0.12	+26.1	0.11	1	+17.5	0.16	4	8.6	−8.6
SP 03				−17.2	0.12	+25.7	0.11	1	+17.6	0.12	4	8.0	−8.5
SV 129	M3	Kotredež, Slovenia (11)	Dfc ‐ ET	−16.9	0.11	+23.3	0.26	1	+14.5	0.17	4	8.8	−12.1
SV 133				−15.9	0.12	+24.9	0.11	1	+16.5	0.06	2	8.5	−9.8
SV 60				−17.1	0.12	+22.0	0.11	2	+12.8	0.01	2	9.2	−14.1
TK 001	I	Termesson National Park, Turkey (12)	Csa	−12.2	0.12	+27.7	0.11	1	+18.8	0.13	3	8.9	−7.1
TK 002 C				−12.2	0.12	+28.0	0.11	1	+19.5	0.08	3	8.5	−6.3
TK 003 C ^				−12.6	0.12	+27.7	0.11	1	+20.5	0.07	2	7.2	−5.1
PP01	M3	Phoenix Park, Ireland (1)	Cfb	−17.1	0.12	+24.2	0.11	1	+15.9	0.11	3	8.4	−10.5
PP02				−16.1	0.12	+25.7	0.11	1	+16.2	0.17	2	9.5	−10.1
PP03				−18.0	0.12	+23.5	0.11	1	+15.2	0.15	2	8.3	−11.3
PP04				−16.2	0.12	+25.9	0.11	1	+17.3	0.15	2	8.6	−8.9
PP05				−17.3	0.12	+24.9	0.11	1	+16.1	0.28	2	8.7	−10.2
PP06				−17.0	0.12	+25.2	0.11	1	+17.1	0.05	2	8.1	−9.0
PP07 *				−16.9	0.12	+23.4	0.11	1	+16.8	0.15	2	6.7	−9.4
PP08				−16.9	0.05	+25.1	0.04	2	+16.6	0.00	2	8.5	−9.6
PP09				−17.2	0.12	+25.0	0.11	1	+16.9	0.01	2	8.1	−9.3
WW001	M3	Wytham Woods, UK (3)	Cfb	−16.1	0.12	+26.9	0.11	1	+17.9	0.00	2	9.0	−8.1
WW002				−15.8	0.12	+25.4	0.11	1	+17.0	0.13	4	8.3	−9.1
WW003				−15.3	0.12	+26.2	0.11	1	+18.1	0.00	2	8.1	−7.9
WW004				−15.8	0.12	+25.8	0.11	1	+18.2	0.17	3	7.6	−7.8
WW005				−14.8	0.12	+28.0	0.11	1	+19.1	0.08	2	8.9	−6.7
WW006				−17.4	0.12	+25.1	0.11	1	+16.5	0.10	2	8.5	−9.7
WW007				−16.8	0.12	+26.0	0.11	1	+18.0	0.01	2	8.0	−7.9
WW008				−13.3	0.12	+26.0	0.11	1	+16.9	0.14	3	9.1	−9.3
WW009				−17.1	0.12	+25.6	0.11	1	+16.7	0.01	2	9.0	−9.6
WW006	I			−16.3	0.12	+26.4	0.11	1	+16.9	0.10	2	9.5	−9.3
WW007				−15.2	0.12	+25.6	0.11	1	+16.7	0.20	2	8.9	−9.5
WW008				−15.2	0.12	+26.5	0.11	1	+17.4	0.10	2	9.1	−8.7
WW009				−16.0	0.12	+26.1	0.11	1	+17.0	0.00	2	9.1	−9.2

### 2.3 Sample preparation

The enamel preparation method for all analyses (cutting and mechanical cleaning) is after Montgomery.^44^ A section of crown surface was abraded to a depth of >100 μm using a tungsten carbide dental bur and the removed material discarded. A thin slice of enamel was then cut from the tooth using a flexible diamond‐edged rotary dental saw.

While sequential sub‐sampling is frequently used to investigate seasonal climatic fluctuations and/or movement of herbivores,^42^, ^45^, ^46^, ^47^ bulk enamel samples were used in this study. These larger sections represent an “average” isotope ratio, which relates indirectly to the average meteoric water δ^18^O value during tooth formation, i.e. a period of several months to several years.^42^ As the year/month of birth, age and year/month/season of death data for many of the wild animals included in this study are unknown, these values provide an average signature for each tooth during formation. To provide consistency across the samples, the same section of tooth was measured in each individual. The most worn 3^rd^ molar from across the sample was measured at 6 mm from the cervix of the crown to tip. Each 3^rd^ molar sample from other individuals was cut to reflect this. None of the 1^st^ incisors showed signs of wear in the same way and so the full length of the tooth was sampled in each case.

All sawn surfaces were mechanically cleaned with a tungsten carbide dental bur, and any adhering dentine was removed. The enamel chips were cleaned ultrasonically for 5 min in high‐purity water and rinsed twice to remove loosely adhered material. This method ensured that any surficial contaminants were removed.

### 2.4 Isotope analysis of oxygen in structural carbonate (δ^18^O_C_ value)

Approximately 3 mg of clean, powdered enamel was loaded into glass vials and sealed with septa. The vials were transferred to a hot block at 90°C on a Multiprep system (GV Instruments, Manchester, UK). The vials were evacuated and four drops of anhydrous phosphoric acid were added. The resultant CO_2_ was collected cryogenically for 14 min and transferred to a GV IsoPrime dual‐inlet mass spectrometer. The isotope ratios are reported as per mil (‰ ^18^O/^16^O) normalised to the PDB scale using an in‐house carbonate reference material KCM (Carrara marble) calibrated against NBS 19 certified reference material. No matrix‐matched internationally recognised standards were used in this investigation as none are commercially available. The δ^18^O_C_ values were then converted into the SMOW scale using the published conversion equation of SMOW = 1.03091 × δ^18^O PDB + 30.91.^48^ The 1σ reproducibility of the KCM reference material for this set of analyses was calculated by analysis of variance (ANOVA), that separates the within‐batch variation from the between‐batch variation.^49^ The results of the ANOVA for within‐batch repeatability for δ^18^O_C_ and δ^13^C_C_ values were ±0.08‰ and ±0.04‰, respectively. The between‐batch reproducibility was statistically insignificant compared with the within‐batch repeatability.

### 2.5 Isotope analysis of oxygen in phosphate (δ^18^O_P_ value)

Small fragments of clean enamel (15–20 mg) were treated to solubilise PO_4_ anions and precipitated as silver phosphate using a method adapted from O'Neil et al.^50^ The fragments of enamel were cleaned in concentrated hydrogen peroxide (AnalaR – NORMAPUR, BDH, Poole, UK) for 24 h to remove organic material and subsequently evaporated to dryness. The samples were then dissolved in 2 M nitric acid (AnalaR – NORMAPUR, BDH) and transferred to clean polypropylene test tubes. Each sample was then treated with 2 M potassium hydroxide (Merck, Darmstadt, Germany) for neutralisation and 2 M hydrofluoric acid (Romil, Cambridge, UK) to remove calcium from the solution by precipitation of calcium fluoride. The samples were centrifuged and the supernatant added to beakers containing ammoniacal silver nitrate solution and heated gently to precipitate silver phosphate. The silver phosphate was filtered, rinsed, dried and weighed into silver capsules for analysis by continuous‐flow isotope ratio mass spectrometry (CF‐IRMS) using the method of Vennemann et al.^51^ The instrument comprises a TC/EA (high‐temperature conversion elemental analyser) coupled to a DeltaPlus XL isotope ratio mass spectrometer via a ConFlo III interface (ThermoFinnigan, Bremen, Germany). Each sample was analysed in triplicate and the results were corrected against a silver phosphate reference material ‘B2207’ (Elemental Microanalysis Ltd, Oakhampton, UK), which has been measured in an inter‐laboratory comparison study and is reported to have a value of 21.7‰. ^18^O/^16^O ratios were measured against the standard Vienna‐Standard Mean Oceanic Water (VSMOW) with an isotope ratio of 2.0052‰.^52^ The within‐batch repeatability for B2207 by ANOVA produced a p‐value of 0.23, while the between‐batch reproducibility was statistically insignificant by comparison.

## 3 RESULTS AND DISCUSSION

The results of the analyses presented in Table 2 demonstrate a wider range of values across the samples than was expected. The δ^18^O_P_ value range lies from 10.9 to 21.7‰; the δ^18^O_C_ values have a range of 20.1–28.6‰; and δ^13^C values range between −17.97 and −10.38‰.

### 3.1 Duplicate reproducibility

The overall sample reproducibility for the δ^18^O_P_, δ^18^O_C_ and δ^13^C_C_ values was determined by ANOVA. In cases with more than one duplicate, a matrix of replicate values (i.e. 3 analyses = matrix of 3; 4 analyses = matrix of 6; and 5 analyses = matrix of 10) was used to calculate the overall sample reproducibility. The within‐sample reproducibility for δ^18^O_C_ values was ±0.11‰ (1σ, *n* = 27 pairs), for δ^13^O_C_ values was ±0.12‰ (1σ, n = 27 pairs) and for δ^18^O_P_ values was ±0.15‰ (1σ, *n* = 118 pairs) based on sample duplicates.

### 3.2 δ^18^O_P_ and δ^18^O_C_ values

**Figure 2:**
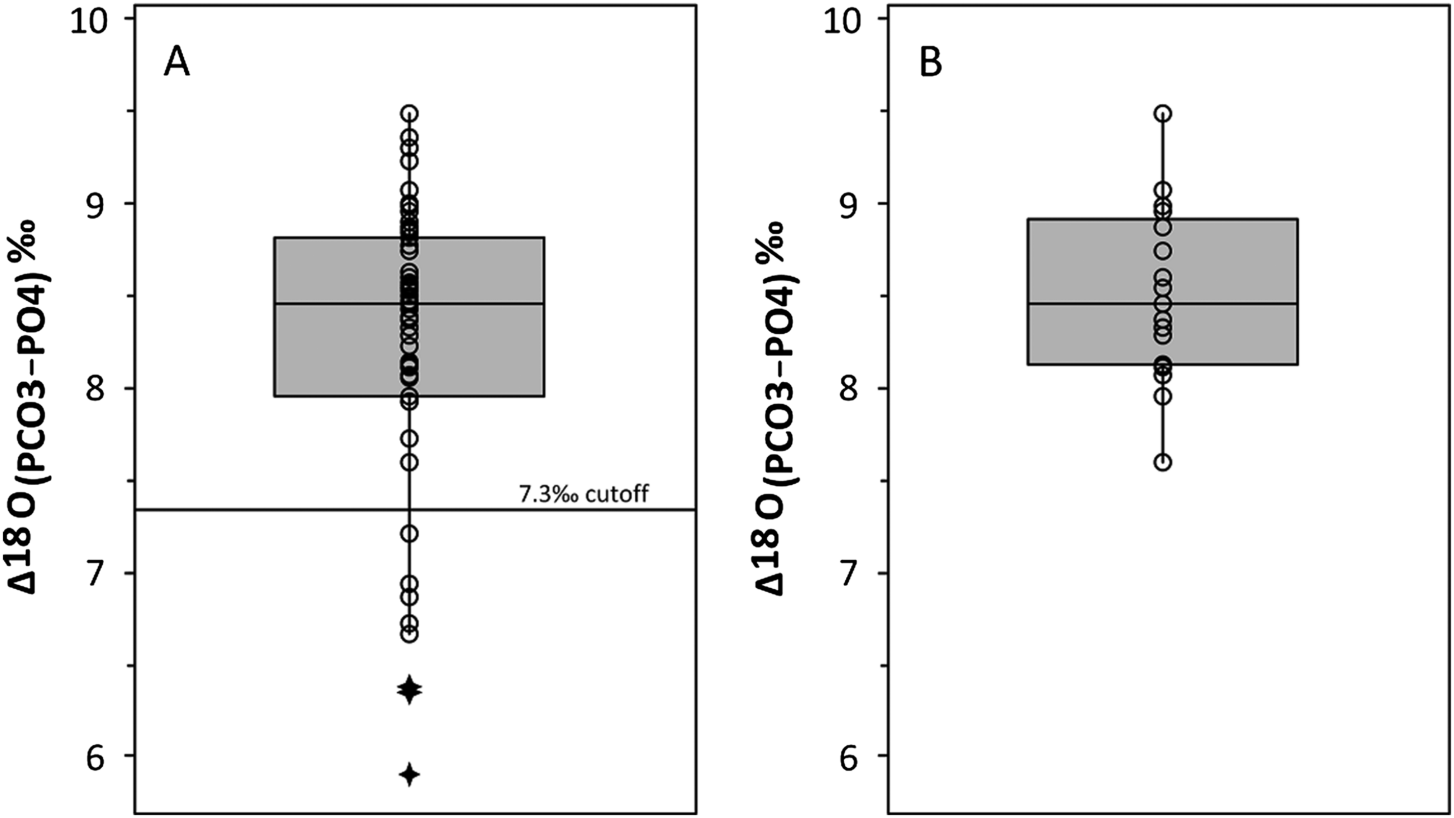
Boxplot showing the variation in Δ^18^O (δ^18^O_C_ – δ^18^O_P_) for modern fallow deer: (A) all fallow data from this study, showing the 7.3‰ cut off for outliers. (B) as (A) but excluding outlier data with values <7.3‰

**Figure 3:**
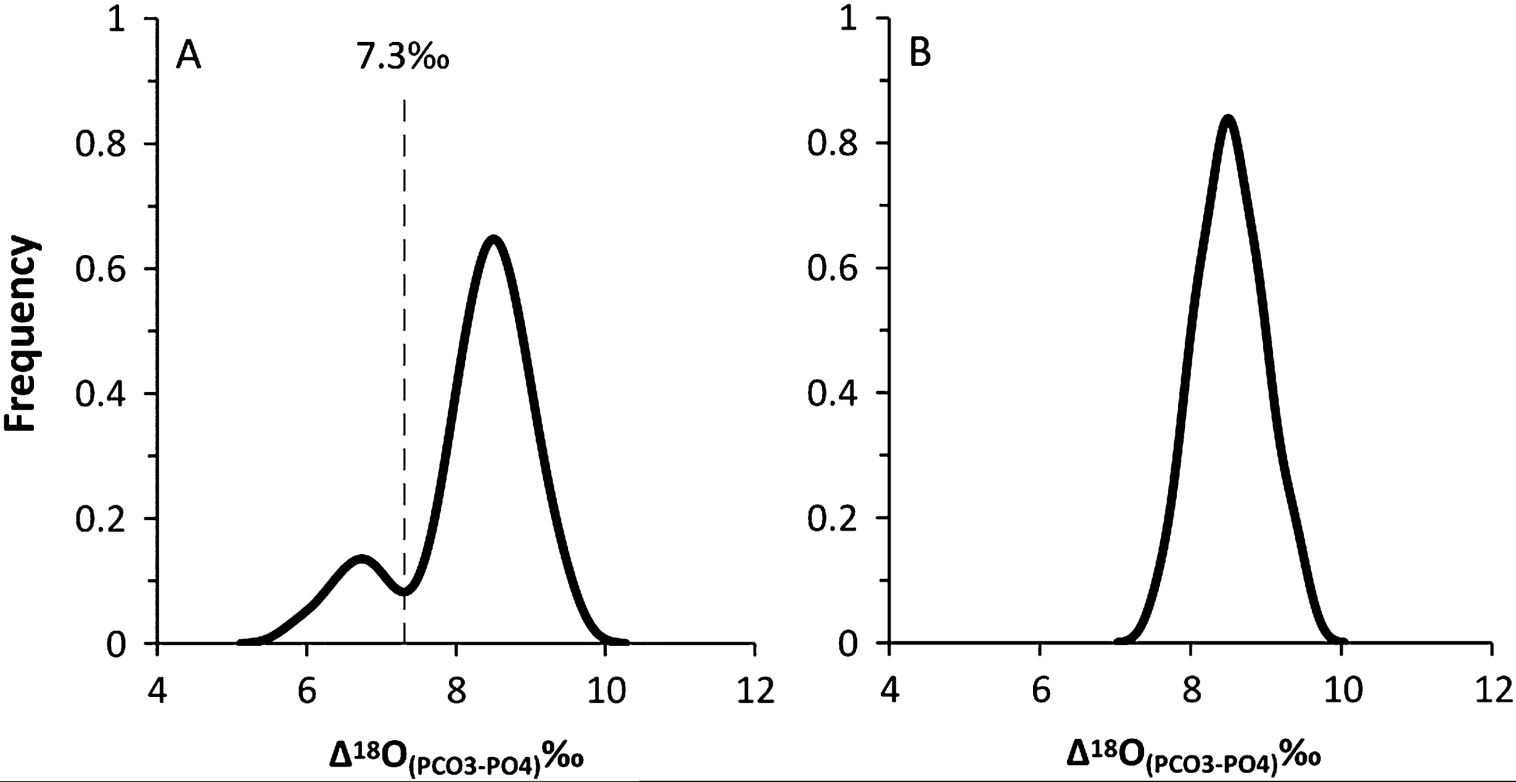
Kernel density plots showing the variation in Δ^18^O (δ^18^O_C_ – δ^18^O_P_) for modern fallow deer: (A) all fallow data from this study, showing the 7.3‰ cut off for outliers. (B) as (A) but excluding outlier data with values <7.3‰

Δ^18^O values represent the difference between the δ^18^O_P_ and δ^18^O_C_ values and are often used to identify evidence of post mortem alteration of enamel or bone phosphate in bio‐apatite.^18^ From the results of this analysis we have an average Δ^18^O value of 8.2 ± 0.8‰, with a range of 5.9–9.5‰. The distribution of the Δ values from this data set is presented in boxplots (Figure 2) and Kernel density diagrams (Figure 3). The boxplot shows that there are three distinct outlying values (samples MV‐004, FR‐02, HA‐645). The Kernel density plot reveals a bimodal distribution. Of the 51 Δ values, 94.1% are normally distributed between 7.6‰ and 9.5‰, and 13.7% have values below 7.6‰. Examination of the Kernel data indicates that the inflection point between the two groups of data is at 7.3‰. There are eight samples with Δ^18^O values <7.3‰, including the three samples identified as outliers in the boxplot (MV‐004, FR‐02, HA‐645, PP‐07, GM‐02, HA‐643, MP‐1, TK‐003C). Previous studies have indicated that the structural carbonate component is more susceptible to alteration than the bone phosphate component,^18^, ^20^ and varying degrees of diagenesis affecting δ^18^O_C_ values in fossil bone and enamel have been noted in the literature.^53^, ^54^ However, in this study of modern tooth enamel, the teeth were collected shortly after death and therefore we do not expect diagenesis to have taken place to any extent. At present we cannot explain the difference in Δ^18^O values for these samples.

### 3.3 The regression

**Figure 4:**
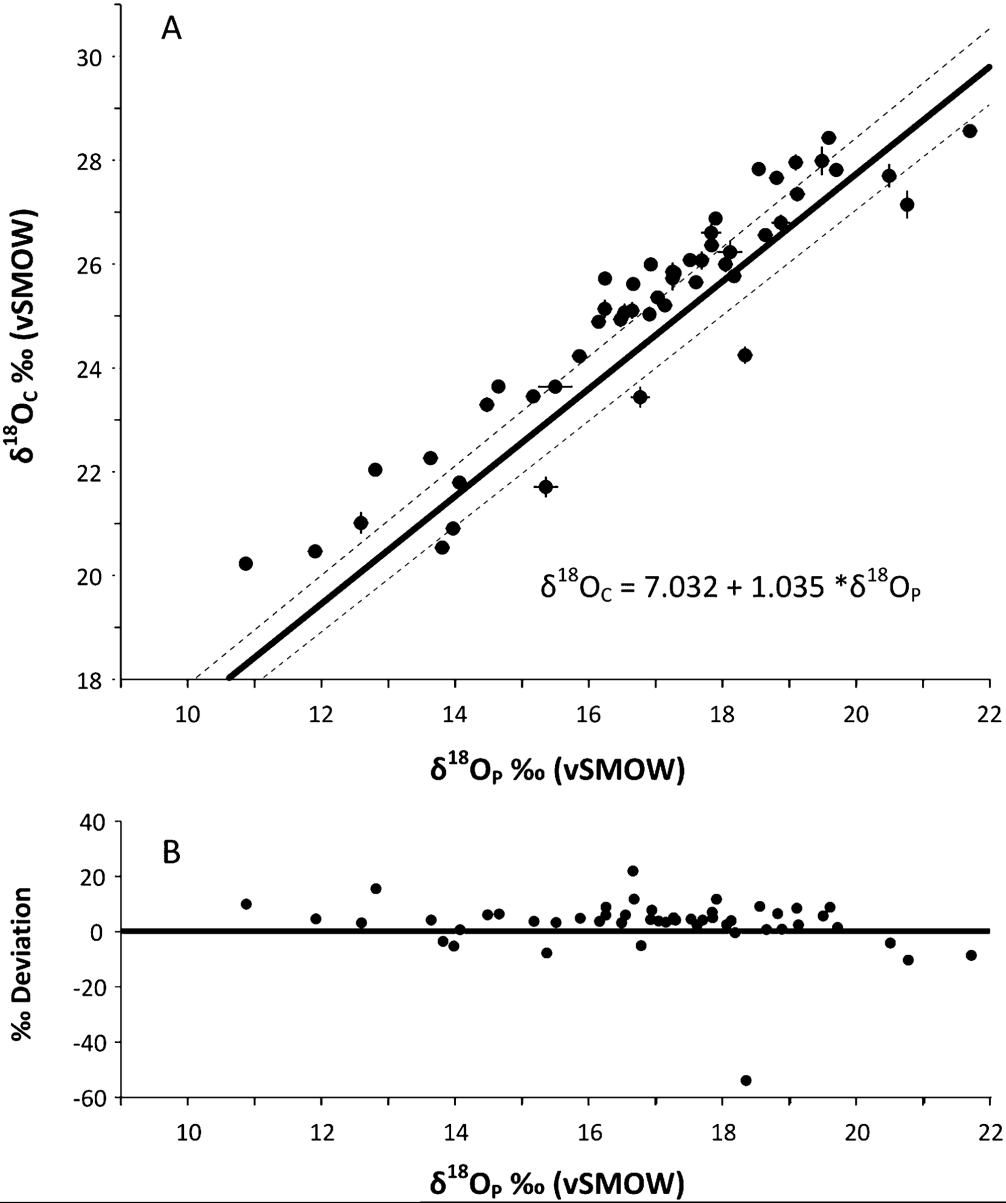
(A) Measured values of structural carbonate oxygen (δ^18^O_C_) and phosphate oxygen (δ^18^O_P_) for all modern fallow deer (51 individuals). FREML line of best fit (δ^18^O_C_ = 7.032 + 1.035 * δ^18^O_P_), and upper and lower 95% confidence limits. (B) δ^18^O_C_‰ deviation from the line of best fit

The linear relationship between phosphate oxygen and structural carbonate oxygen in fallow deer enamel was determined by regressing the data using a Functional Relationship Estimation by Maximum Likelihood (FREML).^55^ FREML regressions take into account errors on both the X and Y variables, whereas standard regressions only allow for errors in the Y variable. The regression carried out on all 51 samples yields the following equations:

$$\delta^{18}O_{C}=+9.244\left(\pm 0.216\right)+0.958^{*}\;\delta^{18}O_{P}\;\left(\pm 0.013\right)$$

and

$$\delta^{18}O_{P}=+0.966\left(\pm 0.010\right)+-6.790^{*}\;\delta^{18}O_{C}\;\left(\pm 0.240\right),$$

where the *p*‐value is 0, r^2^ = 0.8736, the 95% confidence interval is 1.54σ for *n* = 51 and the values within brackets are the standard error (Figure 4).

Our boxplot and kernel density determinations suggest that the eight samples with values <7.3‰ fall outside previously published ranges.^18^, ^56^ In each of these samples the δ^18^O_P_ values are higher than their δ^18^O_C_ values compared with in the other 43 deer (13.8–21.7‰, $\bar{x}$ 17.7 ± 3.4‰, *n* = 8; 10.9–19.7‰, $\bar{x}$ 16.7 ± 2.1‰, *n* = 43 respectively). Therefore, these eight samples have the lowest Δ^18^O values (5.9–7.2, $\bar{x}$ 6.6 ± 0.41‰). The remaining 43 samples have Δ^18^O values ranging from 7.6 to 9.5‰, $\bar{x}$ 8.5 ± 0.44‰, *n* = 43. No relationship has been observed with regard to δ^13^C_C_ values and the outliers compared with the other 43 individuals.

**Figure 5:**
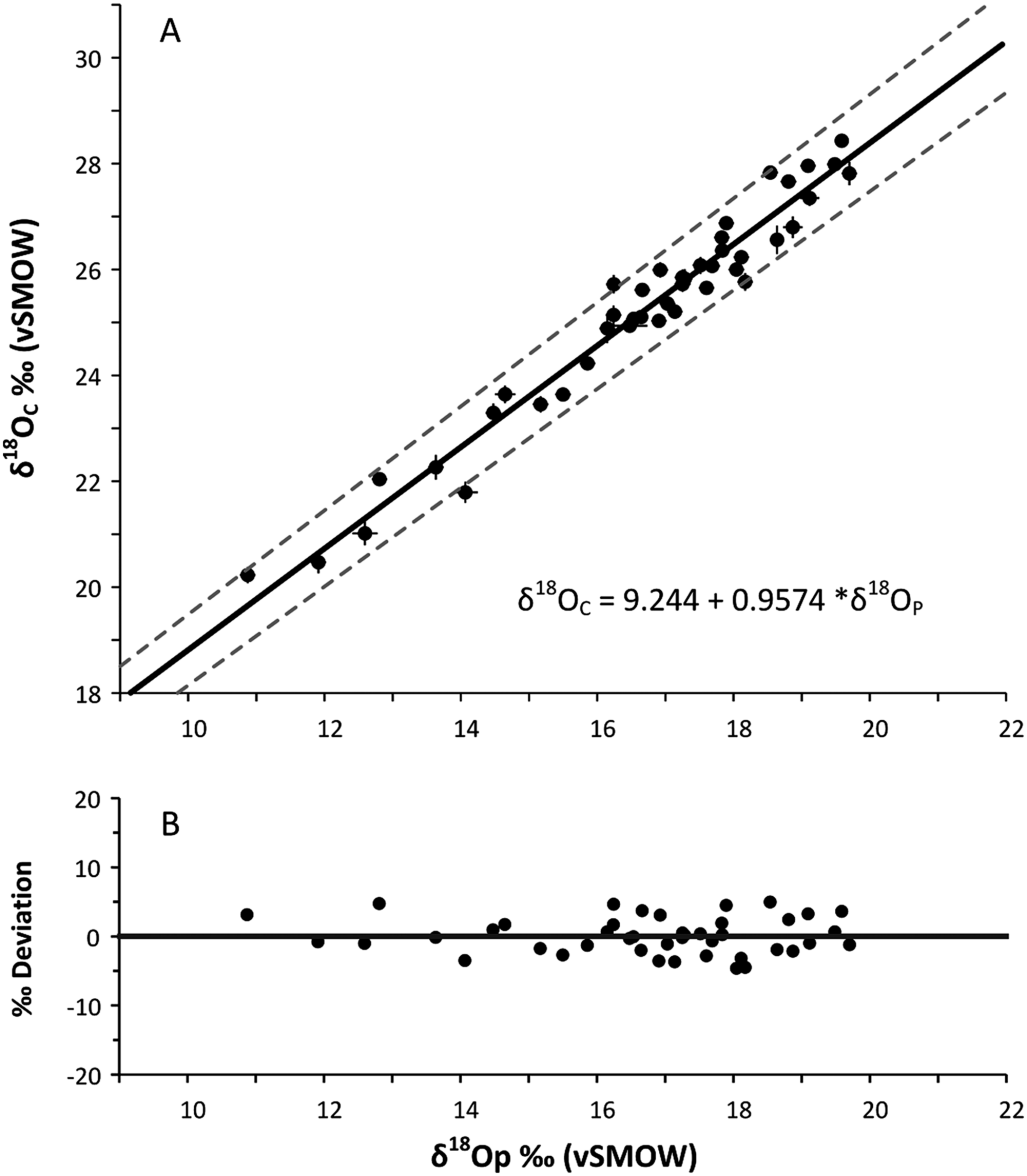
(A) Measured values of structural carbonate oxygen (δ^18^O_C_) and phosphate oxygen (δ^18^O_P_) for modern fallow deer with eight outliers removed (43 individuals, See Figures 2 and 3). FREML line of best fit (δ^18^O_C_ = 9.244 + 0.957 * δ^18^O_P_), and upper and lower 95% confidence limits. (B) δ^18^O_C_‰ deviation from the line of best fit

Results similar to these eight outliers (with low Δ^18^O) were observed by Chenery et al^6^ for human individuals from Egypt, compared with individuals from European locations. They questioned whether this difference was due to a change in metabolic fractionation of the δ^18^O_C_ value as a result of living in a hot arid climate. Although we have limited background data on the deer sampled in this investigation there are no geographic commonalities in terms of habitat and climate zone that would explain the eight fallow deer samples with Δ^18^O less than 7.3‰ as these outliers come from across the sampled populations. At present we do not have an answer for why these samples vary from the regression that encompasses most of our data (43 samples) but it is possible that one might be determined in future. If we remove these eight samples from the data set, the regression of the remaining 43 samples gives the following equations:

$$\delta^{18}O_{C}=+0.957\left(\pm 0.013\right)+9.244^{*}\;\delta^{18}O_{P}\;\left(\pm 0.216\right)$$

and

$$\delta^{18}O_{P}=+1.044\left(+/-0.014\right)+-9.655^{*}\;\delta^{18}O_{C}\;\left(+/-0.351\right),$$

where the *p*‐value is <0.001, r^2^ = 0.956, the 95% confidence interval is 1.39σ for *n* = 43 and the values within brackets are the standard error (Figure 5).

The second regression calculation omitting the eight samples that lay below the regression line has a better correlation coefficient. However, to avoid overlooking potentially important natural variability in fallow deer populations, the rest of the investigation refers to the first regression, determined with all 51 fallow deer specimens included.

### 3.4 Fallow deer and other regressions

**Figure 6:**
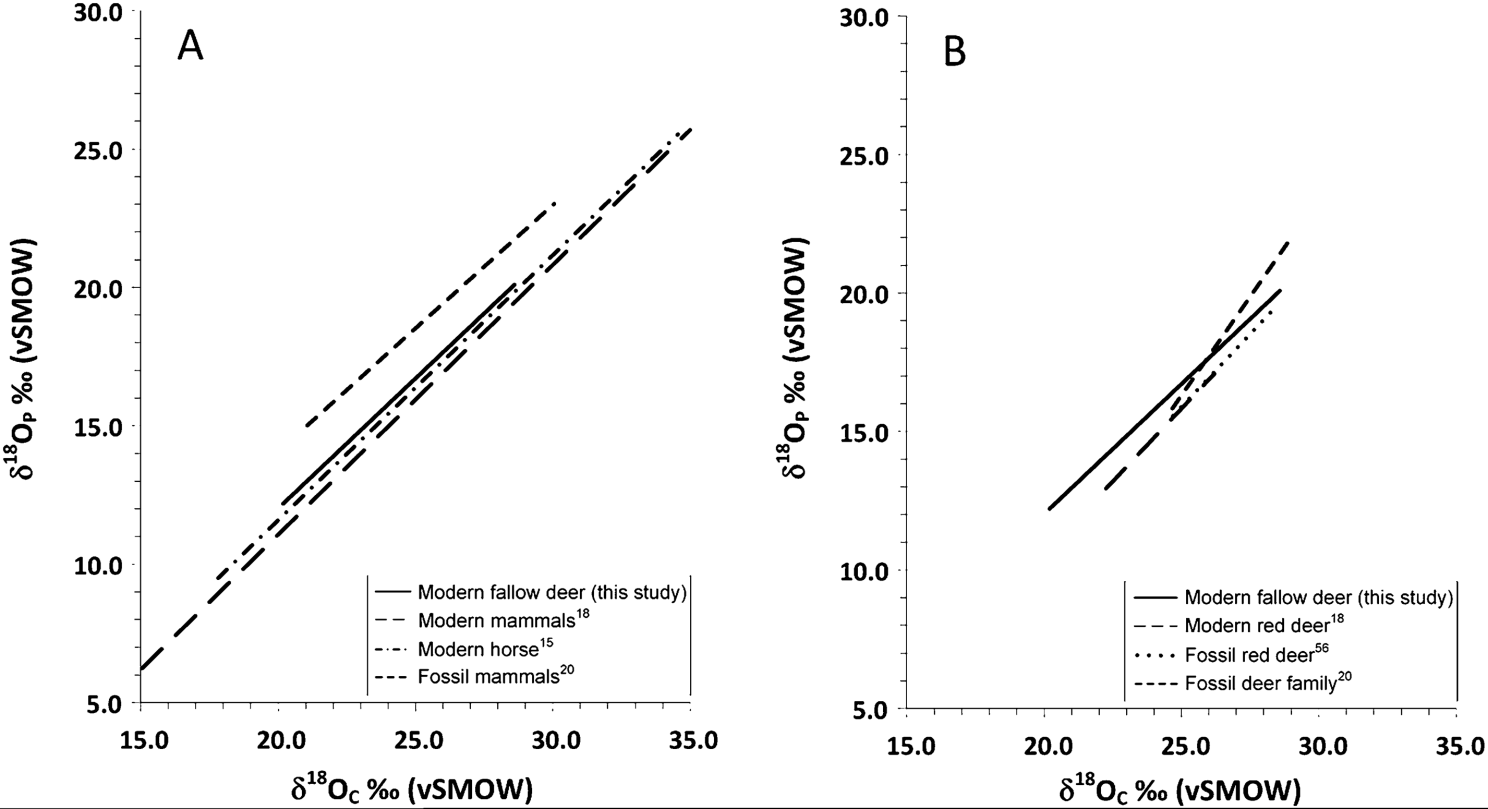
Comparison of published regression data plotted against the regression for fallow deer presented in this paper for (A) other mammals and (B) other cervids

To test whether the fallow deer δ^18^O_P_–δ^18^O_C_ regression differs statistically from those determined for other terrestrial mammals, we compared our regression with correlation equations in major studies by Iacumin et al^18^ for mixed mammals and Bryant et al^15^ for horses (Figure 6A). These data, which cover similarly broad geographic ranges to the fallow deer in this study, have comparable slopes (between 0.963 and 1.044) and variable intercepts (between −7.7‰ and −9.7‰).

Further comparisons show that regression data for mixed fossil species from Germany produced a lower slope of 0.892 and an intercept of −3.788 (r^2^ = 0.66, *n* = 49).^20^ Archaeological human samples from the UK^6^ have correlation equations that also fall within the range of Tütken et al.^20^

No specific studies have previously been carried out on deer species; however, we extracted deer data from larger studies: modern red deer data from Iacumin et al,^18^ Italian fossil red deer data from Pellegrini et al,^57^ and German extinct Miocene deer from Tütken et al.^20^ We used these to calculate the relationship between δ^18^O_P_ and δ^18^O_C_ values for these populations. The results are shown in Figure 6B. The slopes for modern and fossil red deer are very similar to that of modern fallow deer (1.050 and 1.045), which agrees with the findings of D'Angela and Longinelli^34^ who used eight modern fallow deer samples in addition to their red deer regression to suggest that these animals would be similar. The slope for the extinct fossil deer is significantly different at 1.423 which may reflect changes in climatic conditions since the Mid‐Miocene or, perhaps more likely, the alteration of one of the isotopic fractions analysed.^53^, ^54^

### 3.5 Inter‐tooth variation

**Figure 7:**
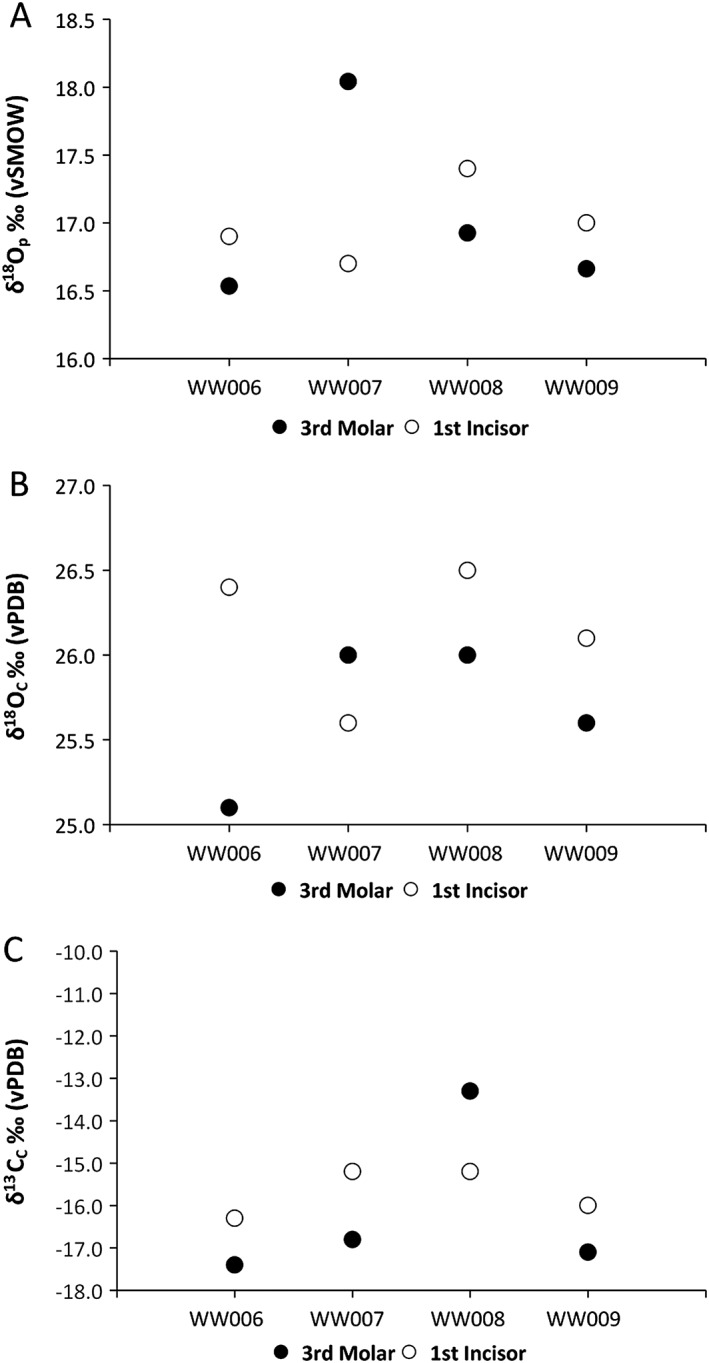
The inter‐tooth variation in 1^st^ incisor and 3^rd^ molar teeth in four individuals from Wytham Woods in (A) δ^18^O_P_, (B) δ^18^O_C_, and (C) δ^13^C values

In the case of four individuals, we were able to examine the differences between 1^st^ incisors and 3^rd^ molar teeth (Table 2 and Figure 7). None of these individuals were deemed outliers in Δ^18^O by our statistical tests. The data show no consistent difference between teeth for δ^18^O_P_, δ^18^O_C_ and δ^13^C_C_ values. This is based on a small sample set; however, the differences between teeth in the same individual are no more or less significant than those seen between each of the three individuals from the 13 geographic locations in this study. This has significant implications for sampling strategy, and while we continue to consider the results from 1^st^ incisors and 3^rd^ molar teeth together in this investigation, we acknowledge that this requires further testing to see if this has further bearing on our results and interpretations.

### 3.6 Fallow deer δ^18^O variation

Despite the successful regression confirming the relationship between δ^18^O_P_ and δ^18^O_C_ values in fallow deer there is considerable variation in the values of δ^18^O in samples from:

1. the same locations
2. samples within modern geopolitical countries
3. samples in neighbouring modern geopolitical countries (see Table 2).

**Figure 8:**
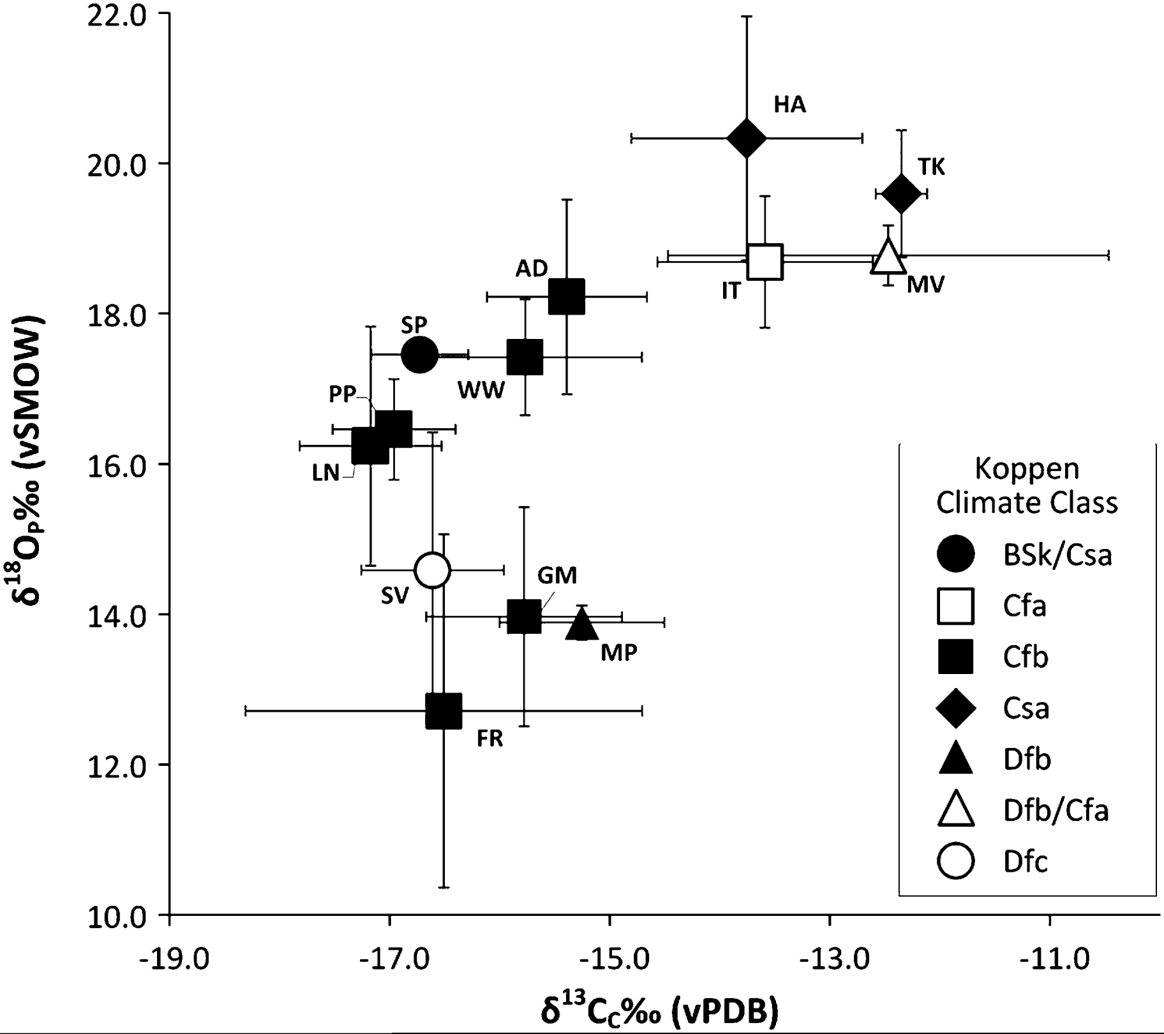
Mean δ^18^O_C_/δ^18^O_P_ values for each location. Error bars represent 1σ of the mean. The locations are: AD = Andover, UK; FR = Gerstheim, France; GM = Sennelager, Germany; HA = Haifa, Israel; IT = Piedmont, Italy; LN = Scrivelsby Park, Lincoln, UK; MP = Moss Park, Norway; MV = Morović, Serbia; SP = Doñana, Spain; SV = Kotredež, Slovenia; TK = Termesson National Park, Turkey; PP = Phoenix Park, Ireland; WW = Wytham Woods, UK. Köppen‐Geiger climate classifications can be found in Table 3, 36

These variations are greater than was expected for animals with the same physiology collected in the same area (see error bars, Figure 8). It was expected that the tooth samples from the same sites, homogenised over several seasons, would give some overlap in values. Studies based in North America have also found discrepancies in the δ^18^O values of biogenetic apatite of white‐tailed deer populations^36^, ^58^ which are likely to be similar in physiology to fallow deer. Their findings suggest that humidity can have an important effect on the δ^18^O composition of deer species.^59^ This means that the relative humidity of habitats, as well as local rainfall patterns, may also need to be considered in evaluations of fallow deer δ^18^O values.

To investigate this further, we attempted a correlation between fallow deer δ^18^O_P_ (SMOW) and δ^18^O_DW_ values from the GNIP database of meteoric water values closest to the sites of the fallow deer populations represented in the study. Although we obtained a linear regression equation (FRMIL) for this (supporting information), the statistics for the equation are poor, particularly the r^2^ value. These results are largely due to:

- the small number of fallow deer investigated at each site
- the unexpectedly wide range of oxygen isotope ratios for each geographic group of deer
- the nearest GNIP data locations rarely corresponding well to the deer locations
- the GNIP data for each location varying in terms of the number of years available, the number of measurements per year and season of collection, and the wide range of δ^18^O values recorded

**Figure 9:**
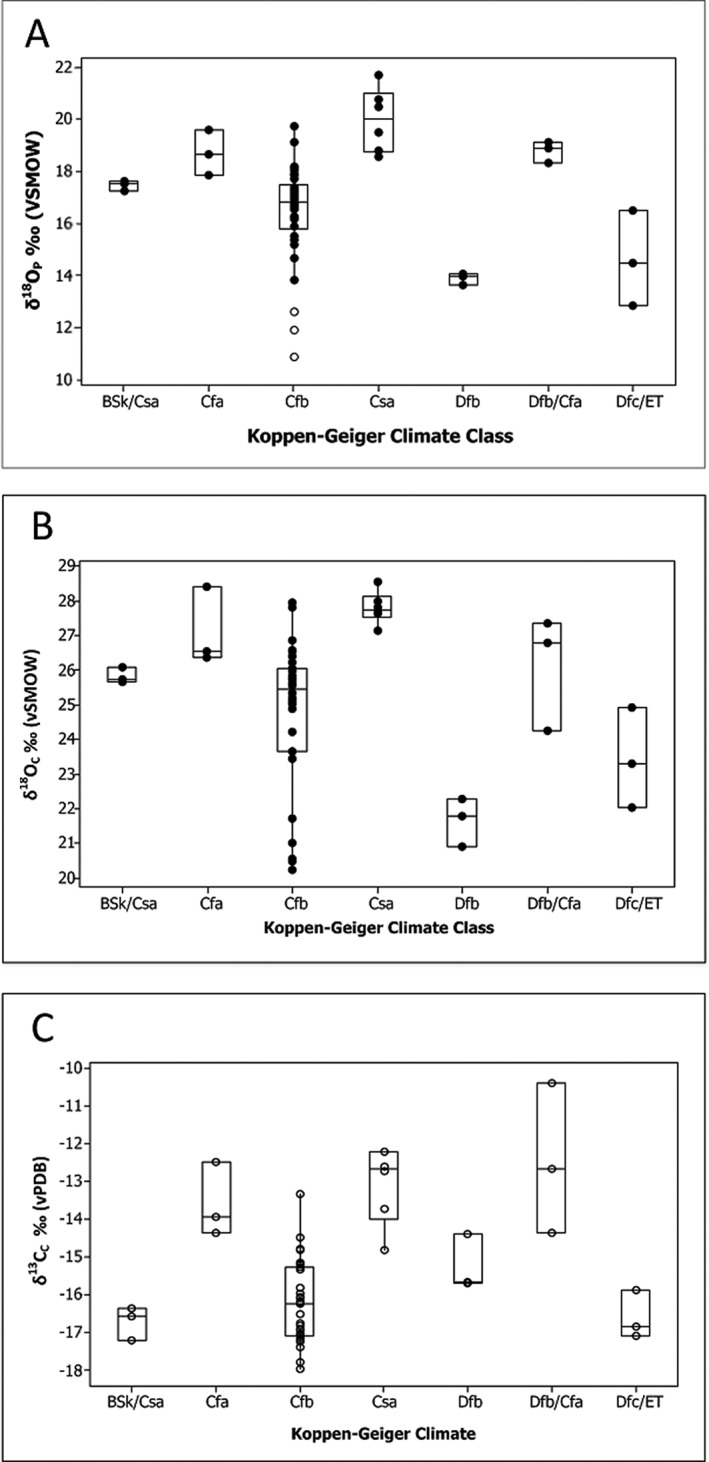
Boxplots of fallow deer (A) δ^18^O_C_, (B) δ^18^O_P_, and (C) δ^13^C results organised by climate classification

Without a reliable drinking water equation, we considered the data according to the climate zones of the sites they were recovered at, according to Koppen‐Geiger classifications (Tables 2 and 3).^60^ We found that fallow deer δ^18^O values cluster well within these climate groupings (Figures 9A and 9B). This means the δ^18^O composition of fallow deer, and other non‐obligate drinking mammals, may be seen as a broader environmental indicator than that of obligate drinking species, which may be more specific in pinpointing provenance based on drinking water. It is important to consider why this may be the case.

**Table 3:** Köppen‐Geiger climate classifications for fallow deer geographic locations^36^

Koppen‐Geiger climate designation	Geopolitical countries	Description
BSk/Csa	Spain	Semi‐arid climate (BSk) of hot Mediterranean temperatures (Csa). Coldest month averaging +0°C, one month's average temperature above 22°C. At least four months averaging above 10°C. Driest month of summer receives less than 30 mm (1.2 in).
Cfa	Italy	Humid subtropical climate; coldest month averaging above 0°C and at least one month's average temperature above 22°C and at least four months averaging above 10°C (50°F). No significant precipitation difference between seasons, no dry months in the summer.
Cfb	UK, France, Germany, Ireland	Temperate oceanic climate; coldest month averaging above 0°C, all months with average temperatures below 22°C, and at least four months averaging above 10°C. No significant precipitation difference between seasons.
Csa	Turkey, Israel	Hot‐summer Mediterranean climate; coldest month averaging above 0°C and at least one month's average temperature above 22°C and at least four months averaging above 10°C. Driest month of summer receives less than 30 mm.
Dfb	Serbia, Norway	Warm‐summer humid continental climate; coldest month averaging below 0°C, all months with average temperatures below 22°C. At least four months averaging above 10°C. No significant precipitation difference between seasons.
Dfc/ET	Slovenia	Mild tundra climate (ET) and coldest month averaging below 0°C and 1–3 months averaging above 10°C (Dfc). No significant precipitation difference between seasons.

In the wild, fallow deer home ranges are thought to be *ca* 9.75 km^2^ in males and 2.1 km^2^ in females^61^ although Chapman and Chapman^35^ have suggested that this can be wider during mating excursions. Within the managed environments represented by the deer in this study, these ranges can be altered or restricted according to different management strategies: fallow deer in a zoo environment will have very different ranges from those in national parks or hunting reserves. Within the more ‘natural’ ranges, such as larger parks and hunting reserves, landscape and relative humidity may vary, particularly with changes in elevation, but it is also likely that plant availability may vary. Some of the individuals in the study, including those in the zoo, are likely to be more intensively managed by regular or supplemental feeding, but whether or the extent to which this happens may depend on climatic conditions in a given season, or may vary in terms of which foods are used.

While received wisdom suggests that fallow deer are primarily grazers, it has been shown that they will feed on many things including browse, fungi and fruits, as they are opportunistic in their foraging habits.^62^, ^63^ In Jackson's^62^ study of fallow deer diet in the New Forest, UK, he observed that feeding patterns changed seasonally, annually and by locality. Studies of diets in Pisa, Italy, and the Blue Mountains of New Zealand showed that in these locations fallow deer were primarily browsers, probably because deciduous and coniferous woods were the dominant habitat/vegetation types in the study areas.^63^, ^64^ Bruno and Apollonio^63^ also suggested that fallow deer diets differed by sex, being richer in species diversity and nutritional value in males requiring increased protein for antler growth and noted a legume contribution to the diet. Nugent^64^ noted that lichens and fungi were prominent components of the diet in beech forest habitats, although less prevalent and therefore not incorporated in hardwood or ‘exotic’ forests. In general, Bruno and Apollonio comment on ‘plasticity in the feeding habits of fallow deer, setting it among the euryphagic species’.^63^ As fallow deer are non‐obligate drinkers, they take up most of their required water from foodstuffs and dew although they will drink when this is not sufficient.^65^, ^66^ As such, a diet that is wide ranging will emphasise inter‐site variation in plant δ^18^O_P_ values. A study by Flanagan et al^67^ has shown that different plant species in an area have variable δ^18^O composition in stem and, to a greater extent, leaf water. This is because turnover time for plant tissues is dependent on the ratio of leaf water to the transpiration rate, and transpiration rate is affected by humidity. Plants with different leaf thickness, stomatal conductance and in environments of variable humidity will result in different δ^18^O values available in the fallow deer diets.

### 3.7 δ^13^C and δ^18^O values in relation to diet, humidity and available water

Further indication that fallow deer δ^18^O values relate to broader climatic conditions is evident from examination of δ^13^C relative to δ^18^O values (Figures 9A–9C). Kohn et al^59^ demonstrated that for modern herbivores within the region of Lake Turkana, Kenya, browsers and mixed feeders (C3 diet) tend to have higher δ^18^O_P_ values than C4 grazers. Their findings suggest that feeding preferences (browsing vs grazing) and drinking habits affect herbivore metabolic responses to habitat changes in humidity and surface water compositions in relation to their isotopic composition.

As expected, fallow deer from the warmest climatic conditions (Turkey, Israel, Serbia and Italy) have higher δ^18^O_P_ values than those specimens from cooler, western Europe (Figure 9A). The δ^13^C values measured in this study also follow broad climatic zonation. The δ^13^C values from enamel agree with a previous study by Miller et al^2^ that shows how δ^13^C values from bone collagen can be a broad scale environmental indicator in fallow deer, and potentially other herbivores, over a wide geographic scale as δ^13^C values are affected by relative plant aridity.^68^, ^69^

## 4 CONCLUSIONS

This study sought to investigate the relationship between oxygen isotope ratios in structural carbonate (CO_3_^2−^) and phosphate (PO_4_^3−^) in European fallow deer bioapatite, to directly relate it to the composition of ingested water, and to further understand the ecology of the species. The regression equation for the relationship between phosphate and structural carbonate oxygen in fallow deer enamel (δ^18^O_C_ = +9.244(±0.216) + 0.958 * δ^18^O_P_ (±0.013)) resulting from this investigation is in line with data from other mammalian species and fits well with those identified in other cervids. As such, the regression identified provides a useful tool for comparison with other herbivore oxygen data of this type and serves as a conversion equation for fallow deer δ^18^O studies.

Attempts to calculate the relationship between δ^18^O_C_ and δ^18^O_DW_ values were less successful, as individual fallow deer living in the same environments have highly variable δ^18^O values, despite samples being homogenised across the enamel growth axis. While δ^18^O values vary within geographic populations, the results broadly relate to the fallow deer herds' climatic circumstances and can be seen to reflect the ecological zones they are found in. This is likely to be because fallow deer are extremely eclectic feeders, for whom changes in humidity affect not only their own metabolic responses, but also have varying effects on individuals' diets. It is possible that this is the reason why δ^18^O_C_ to δ^18^O_DW_ calculations are difficult to determine for other populations of euryphagic species, such as macaques^70^ and white‐tailed deer.^36^, ^58^

As fallow deer have variable δ^18^O values within geographic locations that have a loose relationship to the local drinking water it is unlikely that the species are a good palaeoclimatic indicator. However, their δ^18^O and δ^13^C values both give broad‐brush indications of the climatic areas that populations inhabited when teeth were forming (δ^18^O and δ^13^C_C_ values in tooth enamel) and in their bulk‐dietary ratios (δ^13^C values in bone collagen^2^). As such, isotope investigations of archaeological populations of fallow deer can still be useful indicators of human translocations and a unique human‐animal relationship in development. Indeed, it is likely that the ability of the fallow deer to source highly variable diets from their ranges, i.e. their adaptability and browse‐graze behaviours, is a significant factor in their successful human‐instigated introductions to so many regions across the globe.^35^

### 4.1 Further directions

Our work has highlighted a number of profitable avenues for research that could help to further the understanding of the relationship between the phosphate and structural carbonate fractionation in fallow deer bioapatite, and by extension potentially other non‐obligate drinking, euryphagic species.

1. Our current findings suggest that there is significant inter‐tooth variation in the oxygen isotope ratios of phosphate and structural carbonate fractionation of fallow deer. Systematic investigation of this would be extremely useful and could radically alter interpretation and impact greatly on sampling strategies. It may also suggest further factors affecting variation in δ^18^O values in fallow deer, and ultimately other species.
2. Although we investigated a drinking water correlation it was not the main focus of our study, which was to investigate the relationship between the phosphate and structural carbonate fractionation in fallow deer bioapatite in tooth enamel. Our initial investigation was ‘desk based’, using published GNIP data, and met with little success. Further work, with the specific aim of finding a correlation, could build upon our initial work and again suggest further factors affecting variable δ^18^O values.
3. For both these investigations, analysis of fallow deer populations and samples with known life histories could negate some of the ambiguities in our study. In particular an understanding of the sex, season and year of birth and specific information about an individual's range and diet would be helpful.

## Supporting information

Data S1.Supporting information
