# Supplementary material for: The relationship between the phosphate and structural carbonate fractionation of fallow deer bioapatite in tooth enamel
Source: Rapid Commun Mass Spectrom. 2018 Dec 19;33(2):151–64. doi: 10.1002/rcm.8324 (PMC6859465; doi:10.1002/rcm.8324)
Supplement: Supplementary file 1 — Data S1.Supporting information [file RCM-33-151-s001.docx]

Supplementary Information

An attempt was made to calculate a drinking water equation from the mean δ^18^O_P_ value (SMOW) for each of the fallow deer locations and the mean δ^18^O value of meteoric water for the nearest equivalent GNIP database locations. The data are as follows:

| Code | Location | δ^18^O_P_ sample | 1sd | GNIP location | Mean δ^18^O Local Predipitation | St.dev | Number of samples |
| --- | --- | --- | --- | --- | --- | --- | --- |
| AD | Andover, UK | 18.22 | 1.3 | Wallingford | -6.71 | 2.25 | 369 |
| Fr | France | 12.71 | 2.3 | Karlsruhe | -7.88 | 2.54 | 396 |
| GM | Germany | 13.97 | 1.5 | Bad Salzuflen | -7.70 | 2.24 | 408 |
| HA | Haifa, Israel | 20.33 | 1.6 | Har Kna'an (Tirat Yael) | -6.20 | 1.40 | 93 |
| IT | Italy | 18.69 | 0.9 | Saluggia | -6.13 | 2.72 | 10 |
| LN | Scrivelsby Park, Lincoln, UK | 16.24 | 1.6 | Keyworth | -7.44 | 2.31 | 118 |
| MP | Moss Park, Norway | 13.89 | 0.2 | Forshult (Sweden) | -11.78 | 3.19 | 62 |
| MV | Serbia | 18.77 | 0.4 | Zagreb (Croatia) | -8.80 | 2.90 | 183 |
| PP | Phoenix Park, Ireland | 16.46 | 0.7 | Armagh Observatory | -8.17 | 1.98 | 20 |
| SP | Spain | 17.46 | 0.2 | Almeria Aeropuerto | -4.27 | 2.72 | 90 |
| SV | Slovenia | 14.59 | 1.8 | Ljubljana | -8.71 | 2.90 | 291 |
| TK | Turkey | 19.59 | 0.8 | Antalya | -4.75 | 2.46 | 358 |
| WW | Wytham Woods, UK | 17.61 | 0.9 | Wallingford | -6.71 | 2.25 | 369 |

The resulting calculation for drinking water is **DW = 33.852 +2.337 * δ^18^O_P_ (SMOW)** where the *p*-value is 0, r^2^ = 0.3046, the 95% confidence interval is 1.79σ for n= 51, and the values within brackets are the standard error.

The r^2^ value is very low, a good indicator of how poor the regression is. The 1σ values are very high for meteoric water and the sample sites, as many of the GNIP locations are not close to the fallow deer sites.

A regression was also attempted for individual deer sample values and the mean local GNIP water values, but the results were even less meaningful and have not been included here.
